# Breastfeeding and Active Bonding Protects against Children’s Internalizing Behavior Problems

**DOI:** 10.3390/nu6010076

**Published:** 2013-12-24

**Authors:** Jianghong Liu, Patrick Leung, Amy Yang

**Affiliations:** 1 Department of Family and Community Health, School of Nursing, University of Pennsylvania, Philadelphia, PA 19104, USA; 2 Department of Psychology, Chinese University of Hong Kong, Hong Kong, China; E-Mail: pleung@cuhk.edu.hk; 3 Department of Biostatistics and Epidemiology, University of Pennsylvania, Philadelphia, PA 19104, USA; E-Mail: yyang@mail.med.upenn.edu

**Keywords:** breastfeeding, bonding, attachment, internalizing behaviors

## Abstract

Breastfeeding is associated with numerous health benefits to offspring and mothers and may improve maternal-infant bonding. Ample evidence suggests breastfeeding can improve child neurodevelopment, but more research is needed to establish whether breastfeeding is linked to the development of child psychopathology. This paper aims to explore the effects of both breastfeeding and mother-child interactions on child behavioral outcomes at a later age. Children from the China Jintan Child Cohort Study (*N* = 1267), at age six years old were assessed, along with their parents. Children who were breastfed exclusively for a period of time in the presence of active bonding were compared to those who were breastfed in the absence of active bonding as well as to children who were not exclusively breastfed, with or without active bonding. Results from ANOVA and GLM, using SPSS20, indicate that children who were breastfed and whose mothers actively engaged with them displayed the lowest risk of internalizing problems (mean = 10.01, SD = 7.21), while those who were neither exclusively breastfed nor exposed to active bonding had the least protection against later internalizing problems (mean = 12.79, SD = 8.14). The effect of breastfeeding on internalizing pathology likely represents a biosocial and holistic effect of physiological, and nutritive, and maternal-infant bonding benefits.

## 1. Introduction

Breastfeeding is associated with a wide range of positive health outcomes in children and mothers. For example, a systematic review and meta-analysis of approximately 400 studies found that breastfeeding was related to a reduced risk of acute ear infections, respiratory tract infections, asthma, obesity, type 1 and 2 diabetes mellitus, and childhood leukemia [1]. The production of prolactin and oxytocin during breastfeeding is associated with lower levels of maternal stress and enhanced bonding [2]. Furthermore, early cessation of breastfeeding or not breastfeeding at all has been linked to an increased risk of maternal postpartum depression [1].

One particular outcome of interest is that of cognitive development in breastfed children. Breast milk is rich in vital nutrients, such as essential fatty acids, vitamins, minerals, and amino acids, that are associated with improved cognitive functioning [3], language development [4], and overall neurological development [2,5]. In addition, breastfeeding has been associated with improved mother-infant bonding [6,7]. For instance, early feeding interactions between mother and infant may result in more positive feeding experiences and produce greater maternal sensitivity and responsiveness to infant needs [8].

Although previous studies have indicated a wealth of nutritional, physiological, and cognitive benefits to children from breastfeeding, little has been done on emotional development and regulation. It is known that childhood internalizing disorders, including depression and anxiety, can affect up to about 20% of children and adolescents [9]. They also increase the risk of future psychopathology in adulthood [10]. The identification of readily modifiable factors, such as breastfeeding, that may protect against childhood internalizing behaviors is therefore important. Studies have not yet found a relationship between breastfeeding and behavioral outcomes during early childhood [11,12]. However, there are limited studies conducted in older age groups. Oddy *et al*. found that breastfeeding may be associated with adverse mental health outcomes in early adolescents while Kwok *et al*. found inconsistent associations [13,14]. However, few studies have tested the long term effect of both breastfeeding as well as the mother-infant interaction during feeding on child behavioral outcomes.

The purpose of this study is to examine whether breastfeeding was related to fewer internalizing disorders later in childhood in a large, community-based sample of Chinese children and parents, and to understand whether breastfeeding and active bonding (*i.e.*, verbal interactions during feeding) were associated with the reduced risk of internalizing behaviors. Finally, this study will assess whether there was any breastfeeding duration (or dosage) effect on internalizing behaviors.

## 2. Experimental Section

### 2.1. Participants and Sample Design

The current study was part of a larger population-based community cohort study of 1656 Chinese children (55.5% boys, 44.5% girls) initially recruited from four preschools in the town of Jintan, located in the southeastern coastal region of Mainland China. Briefly, all children and parents taking part in the original cohort study were invited to participate for assessment of children’s behaviors while the children were in the final few months of their senior year in preschool (Spring 2005 to Spring 2007). At that point, some children dropped out of the study because of changing schools or because bio-markers were not obtained originally; therefore, only 1419 children in the original sample were followed up in the later waves. Detailed sampling and research procedures of this larger cohort study have been described elsewhere [15,16].

We excluded the cases where the children are over six years old (there are quite a few because it was late in the school year) for the purpose of this analysis because we are using Child Behavior Checklist (CBCL) 1.5 to 5 to measure behavioral outcome. We performed a comparison on measures such as mother’s age when the children were born, neighborhood problems, gender, parent’s education and occupation prestige, as well as whether parents are separated or divorced, and found there is no difference except the older children tend to be boys and have parents who are less educated. We acknowledge that Chinese tradition prefers to hold boys back in the earlier years of education because of the cultural belief that boys develop and mature later than girls, and that more educated parents would want to place their children in school as early as possible.

The analysis sample consists of 1267 complete data. The mean age of the analysis sample was 66.6 months (SD = 5, range = 50.0–71.9). This is close to the common kindergarten age in the US.

### 2.2. Measures

#### 2.2.1. Internalizing Behavior Problems

We used the Internalizing Behavior scale from the Child Behavior Checklist (CBCL)/1.5-5 as the measure for the dependent variable. The factor structures of CBCL/1.5-5 have been validated in our previous study [17]. The internalizing behavior scale consists of 36 items out of 99 items in total, including Emotionally reactive, Anxious/Depressed, Withdrawn and Somatic Complaints. Items are rated on a 3-point scale (0 = not true, 1 = sometimes true, or 2 = often true) [18]. In this study, we utilized both the full Internalizing Behavior scale and the four syndromes as dependent variables. Alpha is 0.87 for the scale in our sample. We used raw scores on all behavioral assessments for the analysis, as recommended by Achenbach [19].

#### 2.2.2. Breastfeeding

Mothers completed a retrospective questionnaire that asked whether they breastfed (78.3%), used formula (5.6%) or both (16%). In this study, we define exclusive breastfeeding as exclusive for a period of time with a minimum of one month. Non-exclusive breastfeeding is defined to include formula only or mixed methods. Mothers were also asked to report breastfeeding duration in months (mean = 8.79, range 0–24). Duration was categorized into three levels: less or equal to 7 months (25%), between 7 and 10 months (51%) and greater or equal to 10 months (24%).

#### 2.2.3. Maternal Interaction

Mothers were asked a follow-up question for (breast) feeding: “Did you talk to the child while (breast) feeding in the first two years” These responses were: 1 = Never, 2 = Sometimes, 3 = Always. In our study, not all mothers were always talking to the child while breastfeeding. We combined the two measured into one and named it “Feeding types and bonding”, reflecting the beneficial effects of both nutritional and active communication. In our sample, we identified four groups. 34.8% of the mothers who always use breast milk and always talk to the child while feeding were classified into Breastfed and Active bonding (group 1); 43.5% who never talk to the child even when they used breast milk were assigned into Breastfed and no Active Bonding (group 2); 9% of the mothers were in the group of Non-breastfed and Active Bonding (group 3), and the final 12.7% in the Non-breastfed and no Active Bonding (group 4).

#### 2.2.4. Social Adversity

Parents were asked to fill in a sociodemographic questionnaire at the same time they completed the CBCL when children were six years old. A number of researchers have demonstrated the utility of combining of several individual measurements of psychosocial risk factors in studying child behavior problems [20,21]. The adversity index was created along lines similar to those described by Rutter and Moffitt [20,21]. A total adversity score was derived based on 11 variables. This score was created by adding 1 point (for 9 of the 11 indicators) or 2 points (for 2 indicators) for 11 adversity indicators: mother’s low education (below middle school, 8.8%), father’s low education (below middle school, 5.4%), mother’s low occupational status (3-point scale: 0 = professional or skilled work, 22.2%; 1 = un-skilled worker, 38.9%; and 2 = no occupation, 31.8%), father’s low occupational status (3-point scale: 0 = professional or skilled work, 26.8%; 1 = unskilled worker, 60.3%; and 2 = no job, 3.8%), mother’s poor health status (2.1%), father’s poor health status (3.8%), obstetric complication (bleeding, hypertension, diabetes, Caesarian section, difficult birth, low birth weight, difficulty breathing, 35.6%), divorce (3.3%), absence of biological mother (4.2%), house size below 70 m^2^ (8.4%), and poor neighborhood (overcrowded neighborhood, noise pollution, damp, 35.6%). Details on these indicators are given in Liu *et al*. [22]. The adversity score ranged from 0 to 13 (M = 3.51, SD = 2.04).

### 2.3. Statistical Methods

To test whether there is any social adversity or internalizing behavior score differences between exclusive and non-exclusive breastfeeding groups, independent *t* tests were employed. Differences in proportions for gender were tested using χ^2^ tests. The same tests were conducted again to test differences between active bonding and non-active bonding group. Cohen’s *d* was calculated to show the size of the effect between two comparison groups.

To test for the combined effects of feeding and bonding types on internalizing behaviors, a series of analysis of variance (ANOVA) were performed. “Feeding and bonding types” was a new variable (four groups defined in the method section) created from the two independent binary variables. It was the grouping variable in ANOVA, and the dependent variables were emotionally reactive, Anxious/Depressed, Somatic Complaints, Withdrawn and total internalizing problems respectively. Omega squared *w*^2^ was computed as an index of effect size with several groups.

To test for the effect of social adversity as a potential confound, general linear models were fitted with adversity entered as covariate. The effect moderator of sex was assessed by entering the measure as a factor alongside feeding and bonding type. Gender × breastfeeding and bonding types interaction term was included in the model as well. The interaction effect of breastfeeding types and bonding types was tested in the models besides including these two variables independently in the models. To test for a dose-response relationship between breastfeeding duration and internalizing behavior, the grouping variable of duration took on three levels (≤7 months, 7–10 months and ≥10 months) for univariate ANOVAs. All results were considered significant if *P* < 0.05 using a two-tailed test. Statistical analyses were conducted by using SPSS version 20.0 (IBM SPSS Statistics).

## 3. Results

### 3.1. Single Effect of Breastfeeding or Active Bonding

Of the 1267 participants with completed data, 77% exclusively breastfed their babies and 43.4% always talk to their babies while feeding. Mean (SD) scores, effect sizes (Cohen’s *d*) and results of specific *t* test comparisons for the two pairs of comparison groups are given in Table 1. The children who were exclusively breastfed had significantly lower mean scores for somatic complaints and total internalizing problems. Active bonded children had significant lower scores across emotionally reactive, anxious/depressed, withdrawn syndromes and total internalizing problems.

### 3.2. Combined Effect of Breastfeeding and Active Bonding

ANOVA results showed significant group differences on mean behavior scores for total internalizing (*F*(3,1264) = 5.21; *P* = 0.001), anxious/depression (*F*(3,1263) = 2.779; *P* = 0.04), somatic complains (*F*(3,1264) = 3.20; *P* = 0.023) and withdrawn (*F*(3,1264) = 6.75; *P* < 0.001). Emotionally reactive showed borderline significance (*F*(3,1261) = 2.38; *P* = 0.068). Mean (SD) scores, effect sizes (omega squared *w*^2^) and *P* values from ANOVA for the four comparison groups are given in Table 2. Results revealed identical trend across all dependent variables, with group 1 displaying the lowest scores, followed by group 3. Group 4 had the highest scores.

### 3.3. Potential Confounds

Demographic and social variables are known to influence breastfeeding and children’s behavior. Our study showed that boys were more likely be fed by formula or a mixed method rather than pure breast milk (χ^2^ = 2.17; *P* = 0.031). Social adversity scores were significantly lower for those whom actively bonded with their baby while feeding (*t* = 5.18; *P* < 0.001), indicating they had a better socioeconomic background and health status (Table 1). Consequently, it is possible that social adversity and gender could account for the main effects of breastfeeding and bonding. We also tested the correlations between potential confounders and dependent variables. In our sample, Spearman correlation indicates social adversity was positively correlated to each syndrome and total internalizing problems (*P* < 0.001 for all), although no significant correlation was detected between gender and dependent variables. As gender and social adversity were correlated to either the breastfeeding and bonding types or the internalizing problems, we entered these two constructs in to a series of general linear models. The main effects remained significant for Anxious/Depressed, Somatic Complaints, Withdrawn and total internalizing (Table 3) after controlling for adversity (*F*(1,1252) ≥ 10.98; *P* ≤ 0.001 for all) and gender (*F*(1,1252) ≤ 1.56; *P* ≥ 0.212 for all).

### 3.4. Effect Moderator

There was no interaction between breastfeeding and bonding grouping and gender (*F*(1,1252) ≤ 2.1; *P* ≥ 0.097 for all), indicating that this measure did not moderate the effects of breastfeeding and bonding.

### 3.5. Dose Response Relationship

**Figure 1:**
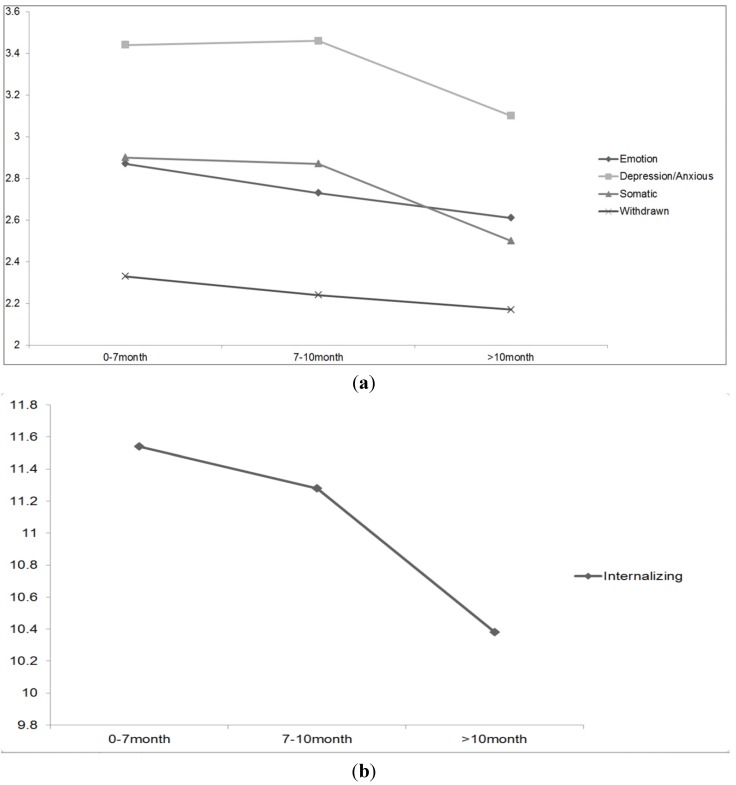
Dose-response Relationship between duration of breastfeeding and internalizing behavior. (**a**) Breastfeeding duration and internalizing syndromes; (**b**) Breastfeeding duration and total internalizing problems.

Univariate ANOVAs (with three groups: 0–7 months, 7–10 months and 10 months duration levers) were conducted on each syndrome and total internalizing scores. Results showed significant main effect for breastfeeding duration on anxious/depression (*F*(2,1170) = 3.28; *P* = 0.038), somatic complains (*F*(2,1171) = 3.25; *P* = 0.039) and total internalizing (*F*(2,1171) = 2.99; *P* = 0.051). No significant dose-response effect was detected for emotionally reactive (*F*(2,1170) = 1.21; *P* = 0.298) and withdrawn (*F*(2,1171) = 0.910; *P* = 0.403). The mean scores for each syndrome and total internalizing problems were plotted against breastfeeding duration levels in Figure 1a,b.

## 4. Discussion

Three key findings emerged from this study. First, compared to children whose mothers breastfed them, children who were not breastfed showed an increased number of internalizing behavioral problems, particularly anxious/depressed and somatic symptoms. Second, the group of children whose mothers both breastfed and actively interacted with their infants had the least likelihood of displaying internalizing problems. Children who were not breastfed but whose mothers still engaged in active interactions displayed the next-lowest risk, while being neither breastfed nor exposed to active bonding had the smallest effect on internalizing behaviors. Finally, a duration effect (dosage effect) appeared such that breastfeeding for 10 months or longer had the strongest impact on reducing anxious/depressed and somatic symptoms in children.

Breastfeeding confers a strong biological benefit to infants and their development [23]. From a nutritive standpoint, breast milk contains docosahexaenoic acid (DHA) omega-3 fats, the consumption of which, along with eicosapentaenoic acid (EPA) fats, may reduce the risk for affective disorders, including major depression and bipolar disorders, particularly among women [24,25]. However, the overall literature on DHA and depression remains mixed [26,27]. What is known, however, is that DHA plays a vital role in neural development, neurotransmitter transmission, and genetic expression, making it highly relevant to child neurodevelopment as well as developmental disorders, such as attention-deficit/hyperactivity disorder and motor deficits [28].

**Table 1:** Descriptives of the Breastfeeding and internalizing behavior Outcome (*N* = 1267).

	Breastfeeding type				Active bonding			
Variables	Exclusive 77%	Non-exclusive 23%	Effect Size (Cohen’s *d*)	*P*	Always 43.4%	Never/Sometimes 56.6%	Effect Size (Cohen’s *d*)	*P*
Emotionally Reactive	2.63 (2.39)	2.85 (2.38)	−0.092	0.168	2.52 (2.30)	2.82 (2.43)	−0.126	0.031
Anxious/Depressed	3.22 (2.28)	3.43 (2.30)	−0.091	0.175	3.13 (2.17)	3.44 (2.36)	−0.136	0.02
Somatic Complaints	2.64 (2.47)	3.01 (2.34)	−0.153	0.025	2.60 (2.33)	2.86 (2.54)	−0.106	0.076
Withdrawn	2.18 (2.34)	2.43 (2.40)	−0.105	0.11	1.89 (2.19)	2.52 (2.44)	−0.271	<0.001
Total Internalizing	10.66 (7.64)	11.7 (7.53)	−0.137	0.04	10.14 (7.05)	11.63 (7.94)	−0.198	0.001
Boy %	53.3	60.4	\	0.031	57.3	53.1	\	0.141
Social Adversity	3.53 (2.03)	3.48 (2.11)	0.0241	0.701	3.17 (1.83)	3.77 (2.15)	−0.104	<0.001

Note: significant results were highlighted in bold; \ not available.

**Table 2:** Combined Effect of Breastfeeding and Active Bonding on Behavior Problems from ANOVA.

	Group 1	Group 2	Group 3	Group 4		
Variables	Exclusive Breastfeeding with Active Bonding 34.8%	Exclusive Breastfeeding without Active Bonding 43.5%	Non-exclusive Breastfeeding with Active Bonding 9%	Non-exclusive Breastfeeding without Active Bonding 12.7%	Effect Size (omega squared *w*^2^)	*P*
Emotionally Reactive	2.48 (2.30)	2.73 (2.45)	2.62 (2.22)	3.07 (2.50)	0.003	0.068
Anxious/Depressed	3.11 (2.17)	3.30 (2.34)	3.15 (2.15)	3.71 (2.41)	0.004	0.04
Somatic Complaints	2.56 (2.39)	2.70 (2.53)	2.72 (2.14)	3.27 (2.50)	0.005	0.023
Withdrawn	1.87 (2.20)	2.40 (2.41)	2.13 (2.22)	2.73 (2.53)	0.013	<0.001
Total Internalizing	10.01 (7.21)	11.12 (7.90)	10.61 (6.66)	12.79 (8.14)	0.01	0.001

Note: significant results were highlighted in bold.

**Table 3:** General linear model statistics of breastfeeding type and bonding against internalizing problems, controlling for gender and social adversity.

Dependent Variables	Type III Sum of Squares	*df*	Mean square	*F*	Significance
Emotionally Reactive	34.4	3, 1255	11	2.045	0.106
Anxious/Depressed	38.12	3, 1257	12.71	2.471	**0.06**
Somatic Complaints	54.6	3, 1258	18.2	3.108	**0.026**
Withdrawn	90.07	3, 1258	32.36	6.016	**<0.001**
Total Internalizing	774.39	3, 1258	258.13	4.573	**0.003**

Note: significant results (*P*<0.05) were highlighted in bold.

It may be that the biological benefit offered to breastfed infants plays a role in healthy cognitive maturation which in turn lowers their risk for psychopathology [29]. Feldman and Eidelman report that breastfeeding is associated with improved motor and social skills [5], but other authors have not found an impact on emotional regulation and behavioral disruption, indicating the need for further research on breastfeeding and child psychodevelopment [30]. Interestingly, a recent study examined effects of breastfeeding on mental health outcomes among children at age 14 years and found that breastfeeding at age six months was associated with a lower rate of child psychopathology, including social and attention difficulties and aggression [31]. However, more longitudinal data is needed to better understand the potential long-term benefits of breastfeeding to child mental health. Whether the nutritional, physiological and cognitive benefits from breastfeeding directly enhance mental health via a biological route in our brain may still require further exploration. However, speculatively, the reported nutritional, physiological and cognitive benefits can confer a lot of advantages to a child to negotiate with the challenges of growing up. For example, a healthier, energetic physical body and a faster cognitive growth can help a child to cope with the arduous demands of modern-day schooling, particularly in mainland China, the tradition of which has long emphasized education as the avenue for upward socio-economic migration. A child who excels at school will also be well liked and accepted by parents, relatives, teachers and peers. School success and social popularity are both known key precursors to mental health [32]. Thus, it is likely that there may be both a direct biological route and an indirect psychosocial route from breastfeeding leading to positive mental health or fewer internalizing problems. These dual routes should both be further examined in future study.

Breastfeeding also provides a biological benefit to the mother by reducing blood pressure and pain [33]. Furthermore, the release of hormones oxytocin and prolactin not only confer analgesic and relaxation benefits, but they also appear to play a key role in mother-infant bonding [11,33], which has been shown to reduce emotional and behavior problems in children [6,7]. 

The attachment aspect of breastfeeding underscores the need to consider its potential mental health benefits. Psychologically, breastfeeding may enhance the mother-infant bonding process via active talking, eye contact, and skin-to-skin touch. This may help mothers form stronger attachments to offspring and improve maternal sensitivity [33], reduce postpartum depression [34], and ward off other negative mood states like maternal stress [35]. This may indirectly benefit a child’s mental health, as the literature detailing the impact of maternal depression on increasing the risk of future child and adolescent psychopathology is compelling [36,37].

Infants may similarly derive a mental health benefit from being breastfed, including development of more secure attachments and reduced negative temperament [38]. Several authors have documented analgesic properties of breast milk, along with reductions in salivary cortisol, due to milk odor and skin-to-skin contact [39,40]; these are hypothesized to help alleviate child distress and strengthen bonding. 

Taken together, these findings underlie a biopsychological aspect of breastfeeding wherein the physiological benefits of breastfeeding (e.g., pain reduction, stress reduction, healthy cognitive development) coupled with improved pair bonding and mother-infant attachments may provide protective effects against the formation of child internalizing behaviors The biopsychosocial interaction may also provide indirect benefits that operate through mediating or moderating variables [41]. For example, secure parent-child attachment may improve child sleep quality [42], and reduced sleep problems in children has been linked to better emotional and behavioral functioning [43]. In addition, breastfeeding is ultimately a holistic process and there are several aspects that facilitate the process, including how the mother responds to the infant, the physical and social environment around the mother and baby, and the level nutrients in the breast milk. These factors, such as the genetic and environmental influences of nutrient intake (e.g., breast milk) should be considered [44]. Consideration of such in larger samples will require further study before definitive conclusions can be drawn about intervening variables on breastfeeding and internalizing conditions.

In this study, breastfeeding for a longer duration (at least 10 months) had the greatest effect on reducing internalizing symptoms. This is consistent with other authors who report that longer duration of breastfeeding was associated with greater protection against child mental health problems at age five years [45]. Another recent longitudinal cohort study from Oddy and colleagues followed breastfed infants to 14 years of age and found that breastfeeding for six months or less independently predicted greater externalizing and internalizing problems in childhood and adolescence, compared to infants who were breastfed for 6 months duration or longer [14]. 

Some important limitations on the present study’s findings exist. First, the use of retrospective data may involve recall bias. However, in the current literature, it is not rare for studies examining breastfeeding practices to use maternal recall data after much longer periods. For example, a study by Promislow looked at maternal recall of breastfeeding duration of elderly US women from 34 to 50 years ago [46]. Nevertheless, future prospective designs should be considered. As previously mentioned, we also did not include holistic measures in our assessment of breastfeeding as we do not have data available due to the retrospective nature of this study. Instead, we included confounding factors, such as demographic and social background, in our analysis of breastfeeding. Another limitation of the present study is that it does not take into consideration the exact duration of exclusive breastfeeding, which is particularly important given the fact that breastfeeding practices have decreased in recent years, especially among urban and well-educated mothers [47]. Future studies should test if there is a duration-dependent relationship between breastfeeding and internalizing behavior in children. Additionally, as active bonding was one of the key predictors, future studies should employ validated, empirical-based measures on this construct. However, despite the use of such “crude” measures, they are able to produce consistent results, indicating evidently the benefits of breastfeeding on the children’s mental health. Furthermore, future studies should stratify by region, given that breastfeeding practices differ by location.

## 5. Conclusions

These results indicate that children who were breastfed and exposed to active bonding during feeding displayed the lowest risks of internalizing behavior problems at age six years. Increased duration of breastfeeding (≥10 months) could also help lower internalizing problems in children (*i.e.*, a dosage effect). These findings were independent of several socio-demographic/family characteristics, as well as gender. It is possible that both nutrients (e.g., fatty acids) and maternal bonding interactively work to promote optimal neurodevelopment in early childhood, subsequently protecting children from internalizing disorders, such as depression, anxiety, and somatic complaints [48]. The promotion of active bonding practices during feeding (whether breastfeeding or formula feeding) may help reduce later internalizing behaviors in children by enhancing attachment between the mother and infant.

## References

[1] Ip S., Chung M., Raman G., Chew P., Magula N., Devine D., Trikalinos T., Lau J. (2007). Breastfeeding and maternal and infant health outcomes in developed countries. Evid. Rep. Technol. Assess..

[2] Uauy R., de Andraca I. (1995). Human milk and breast feeding for optimal mental development. J. Nutr..

[3] Quinn P.J., O’Callaghan M., Williams G.M., Najman J.M., Andersen M.J., Bor W. (2001). The effect of breastfeeding on child development at 5 years: A cohort study. J. Paediatr. Child Health.

[4] Whitehouse A.J., Robinson M., Li J., Oddy W.H. (2011). Duration of breastfeeding and language ability in middle childhood. Paediatr. Perinat. Epidemiol..

[5] Feldman R., Eidelman A.I. (2003). Direct and indirect effects of breast milk on the neurobehavioral and cognitive development of premature infants. Dev. Psychobiol..

[6] Zetterstrom R. (1999). Breastfeeding and infant-mother interaction. Acta Paediatr. Suppl..

[7] Fergusson D.M., Woodward L.J. (1999). Breast feeding and later psychosocial adjustment. Paediatr. Perinat. Epidemiol..

[8] DeWitt S.J., Sparks J.W., Swank P.B., Smith K., Denson S.E., Landry S.H. (1997). Physical growth of low birthweight infants in the first year of life: Impact of maternal behaviors. Early Hum. Dev..

[9] Costello E.J., Mustillo S., Erkanli A., Keeler G., Angold A. (2003). Prevalence and development of psychiatric disorders in childhood and adolescence. Arch. Gen. Psychiatry.

[10] Kovacs M., Devlin B. (1998). Internalizing disorders in childhood. J. Child Psychol. Psychiatry.

[11] Kramer M.S., Fombonne E., Igumnov S., Vanilovich I., Matush L., Mironova E., Bogdanovich N., Tremblay R.E., Chalmers B., Zhang X. (2008). Effects of prolonged and exclusive breastfeeding on child behavior and maternal adjustment: Evidence from a large, randomized trial. Pediatrics.

[12] Papp L.M. (2013). Longitudinal associations between breastfeeding and observed mother-child interaction qualities in early childhood. Child Care Health Dev..

[13] Kwok M.K., Leung G.M., Schooling C.M. (2013). Breast feeding and early adolescent behaviour, self-esteem and depression: Hong Kong’s “Children of 1997” birth cohort. Arch. Dis. Child..

[14] Oddy W.H., Kendall G.E., Li J., Jacoby P., Robinson M., de Klerk N.H., Silburn S.R., Zubrick S.R., Landau L.I., Stanley F.J. (2010). The long-term effects of breastfeeding on child and adolescent mental health: A pregnancy cohort study followed for 14 years. J. Pediatr..

[15] Liu J., McCauley L.A., Zhao Y., Zhang H., Pinto-Martin J. (2010). Cohort profile: The China Jintan child cohort study. Int. J. Epidemiol..

[16] Liu J., McCauley L., Leung P., Wang B., Needleman H., Pinto-Martin J. (2011). Community-based participatory research (CBPR) approach to study children’s health in China: Experiences and reflections. Int. J. Nurs. Stud..

[17] Liu J., Cheng H., Leung P.W. (2011). The application of the preschool child behavior checklist and the caregiver-teacher report form to mainland Chinese children: Syndrome structure, gender differences, country effects, and inter-informant agreement. J. Abnorm. Child Psychol..

[18] Achenbach T., Rescorla L. (2000). Manual for the ASEBA Preschool Forms & Profiles.

[19] Achenbach T.M. (1991). Manual for the Child Behavior Checklist/4–18 and 1991 Profile.

[20] Rutter M., Hersov L.A., Berger M., Shaffer D. (1978). Family, Area and School Influences in the Genesis of Conduct Disorders: Aggression and Anti-Social Behaviour in Child-Hood and Adolescence.

[21] Moffit T.E. (1990). Juvenile delinquency and attention deficit disorder: Boys’ developmental trajectories from age 3 to age 15. Child Dev..

[22] Liu J., Portnoy J., Raine A. (2012). Association between a marker for prenatal testosterone exposure and externalizing behavior problems in children. Dev. Psychopathol..

[23] Rautava S., Walker W.A. (2009). Academy of breastfeeding medicine founder’s lecture 2008: Breastfeeding—An extrauterine link between mother and child. Breastfeed Med..

[24] McNamara R.K., Jandacek R., Rider T., Tso P., Dwivedi Y., Pandey G.N. (2010). Selective deficits in erythrocyte docosahexaenoic acid composition in adult patients with bipolar disorder and major depressive disorder. J. Affect. Disord..

[25] Colangelo L.A., He K., Whooley M.A., Daviglus ML., Liu K. (2009). Higher dietary intake of long-chain omega-3 polyunsaturated fatty acids are inversely associated with depressive symptoms in women. Nutrition.

[26] Makrides M., Gibson R.A., McPhee A.J., Yelland L., Quinlivan J., Ryan P. (2010). Effect of DHA supplementation during pregnancy on maternal depression and neurodevelopment of young children: A randomized controlled trial. JAMA.

[27] Murakami K., Miyake Y., Sasaki S., Tanaka K., Arakawa M. (2010). Fish and *n*-3 polyunsaturated fatty acid intake and depressive symptoms: Ryukyus child health study. Pediatrics.

[28] Kennedy D.O., Jackson P.A., Elliot J.M., Scholey A.B., Robertson B.C., Greer J., Tiplady B., Buchanan T., Haskell C.F. (2009). Cognitive and mood effects of 8 weeks’ supplementation with 400 mg or 1000 mg of the omega-3 essential fatty acid docosahexaenoic aicid (DHA) in healthy children aged 10–12 years. Nutr. Neurosci..

[29] Kramer M.S., Aboud F., Mironova E., Vanilovich I., Platt R.W., Matush L., Igumnov S., Fombonne E., Bogdanovich N., Ducruet T. (2008). Breastfeeding and child cognitive development: A new evidence from a large randomized trial. Arch. Gen. Psychiatry.

[30] Hayatbakhsh M.R., O’Callaghan M.J., Bor W., Williams G.M., Najman J.M. (2012). Association of breastfeeding and adolescents’ psychopathology: A large prospective study. Breastfeed Med..

[31] Rutter M., Rutter M. (1993). Developing Minds: Challenge and Continuity Across the Life Span.

[32] Gribble K.D. (2006). Mental health, attachment and breastfeeding: Implications for adopted children and their mothers. Int. Breastfeed J..

[33] Britton J.R., Britton H.L., Gronwaldt V. (2006). Breastfeeding, sensitivity, and attachment. Pediatrics.

[34] Field T., Diego M., Hernandez-Reif M., Figueirdo B., Ezell S., Siblalingappa V. (2010). Depressed mothers and infants are more relaxed during breastfeeding *versus* bottlefeeding interactions: Brief report. Infant Behav. Dev..

[35] Groër M.W. (2005). Differences between exclusive breastfeeders, formula-feeders, and controls: A study of stress, mood, and endocrine variables. Biol. Res. Nurs..

[36] Rahman A., Harrington R., Bunn J. (2002). Can maternal depression increase infant risk of illness and growth impairment in developing countries?. Child Care Health Dev..

[37] Hay D.F., Pawlby S., Waters C.S., Sharp D. (2008). Antepartum and postpartum exposure to maternal depression: Different effects on different adolescent outcomes. J. Child Psychol. Psychiatry.

[38] Niegel S., Ystrom E., Hagtvet K.A., Vollrath M.E. (2008). Difficult temperament, breastfeeding, and their mutual prospective effects: The Norwegian Mother and Child Cohort Study. JDBP.

[39] Nishitani S., Miyamura T., Tagawa M., Sumi M., Takase R., Doi H., Moriuchi H., Shinohara K. (2009). The calming effect of a maternal breast milk odor on the human newborn infant. Neurosci. Res..

[40] Gray L., Watt L., Blass E.M. (2000). Skin-to-skin contact is analgesic in healthy newborns. Pediatrics.

[41] Liu J. (2011). Early health risk factors for violence: Conceptualization, review of the evidence, and implications. Aggress. Violent Behav..

[42] Vaughn B.E., El-Sheikh M., Shin N., Elmore-Staton L., Krzysik L., Monteiro L. (2011). Attachment representations, sleep quality and adaptive functioning in preschool age children. Attach. Hum. Dev..

[43] Liu J., Zhou G., Wang J., Ai Y., Pinto-Martin J., Liu X. (2012). Sleep problems, fatigue, and cognitive performance in Chinese kindergarten children. J. Pediatr..

[44] Liu J., Tuvblad C., Raine A., Baker L. (2013). Genetic and environmental influences on nutrient intake. Genes Nutr..

[45] Robinson M., Oddy W., Li J., Kendall G.E., de Klerk N.H., Silburn S.R., Zubrick S.R., Newnham J.P., Stanley F.J., Mattes E. (2008). Pre- and postnatal influences on preschool mental health: A large-scale cohort study. J. Child Psychol. Psychiatry.

[46] Promislow J., Gladen B., Sandler D. (2005). Maternal recall of breastfeeding duration by elderly women. Am. J. Epidemiol..

[47] Liu J., Shi Z., Spatz D., Loh R., Sun G., Grisso J. (2013). Social and demographic determinants for breastfeeding in a rural, suburban and city area of South East China. Contemp. Nurse.

[48] Liu J., Chen X., Lewis G. (2011). Childhood internalizing behavior: Analysis and implications. J. Psychiatr. Ment. Health Nurs..

